# Microbiome Alterations Driven by *Trypanosoma cruzi* Infection in Two Disjunctive Murine Models

**DOI:** 10.1128/spectrum.00199-23

**Published:** 2023-05-04

**Authors:** Sergio Castañeda, Marina Muñoz, Peter J. Hotez, Maria Elena Bottazzi, Alberto E. Paniz-Mondolfi, Kathryn M. Jones, Rojelio Mejia, Cristina Poveda, Juan David Ramírez

**Affiliations:** a Centro de Investigaciones en Microbiología y Biotecnología-UR (CIMBIUR), Facultad de Ciencias Naturales, Universidad del Rosario, Bogotá, Colombia; b Department of Pediatrics, Division of Tropical Medicine, Baylor College of Medicine, Houston, Texas, USA; c Texas Children’s Hospital Center for Vaccine Development, Baylor College of Medicine, Houston, Texas, USA; d Department of Molecular Virology and Microbiology, Baylor College of Medicine, Houston, Texas, USA; e Department of Biology, Baylor University, Waco, Texas, USA; f Molecular Microbiology Laboratory, Department of Pathology, Molecular and Cell-Based Medicine, Icahn School of Medicine at Mount Sinai, New York, New York, USA; g Incubadora Venezolana de la Ciencia, Barquisimeto, Venezuela; Hubei University of Medicine

**Keywords:** Chagas disease, MAG, microbiome, *Trypanosoma cruzi*

## Abstract

Alterations caused by Trypanosoma cruzi in the composition of gut microbiome may play a vital role in the host-parasite interactions that shapes physiology and immune responses against infection. Thus, a better understanding of this parasite-host-microbiome interaction may yield relevant information in the comprehension of the pathophysiology of the disease and the development of new prophylactic and therapeutic alternatives. Therefore, we implemented a murine model with two mice strains (BALB/c and C57BL/6) to evaluate the impact of Trypanosoma cruzi (Tulahuen strain) infection on the gut microbiome utilizing cytokine profiling and shotgun metagenomics. Higher parasite burdens were observed in cardiac and intestinal tissues, including changes in anti-inflammatory (interleukin-4 [IL-4] and IL-10) and proinflammatory (gamma interferon, tumor necrosis factor alpha, and IL-6) cytokines. Bacterial species such as Bacteroides thetaiotaomicron, Faecalibaculum rodentium, and Lactobacillus johnsonii showed a decrease in relative abundance, while Akkermansia muciniphila and Staphylococcus xylosus increased. Likewise, as infection progressed, there was a decrease in gene abundances related to metabolic processes such as lipid synthesis (including short-chain fatty acids) and amino acid synthesis (including branched-chain amino acids). High-quality metagenomic assembled genomes of L. johnsonii and A. muciniphila among other species were reconstructed, confirming, functional changes associated with metabolic pathways that are directly affected by the loss of abundance of specific bacterial taxa.

**IMPORTANCE** Chagas disease (CD) is caused by the protozoan Trypanosoma cruzi, presenting acute and chronic phases where cardiomyopathy, megaesophagus, and/or megacolon stand out. During the course of its life cycle, the parasite has an important gastrointestinal tract transit that leads to severe forms of CD. The intestinal microbiome plays an essential role in the immunological, physiological, and metabolic homeostasis of the host. Therefore, parasite-host-intestinal microbiome interactions may provide information on certain biological and pathophysiological aspects related to CD. The present study proposes a comprehensive evaluation of the potential effects of this interaction based on metagenomic and immunological data from two mice models with different genetic, immunological, and microbiome backgrounds. Our findings suggest that there are alterations in the immune and microbiome profiles that affect several metabolic pathways that can potentially promote the infection’s establishment, progression, and persistence. In addition, this information may prove essential in the research of new prophylactic and therapeutic alternatives for CD.

## INTRODUCTION

The flagellate protozoan Trypanosoma cruzi is the causative agent of Chagas disease, a neglected tropical disease endemic in Latin America that affects eight million people worldwide, causing about 12,500 deaths per year (1–4). Chagas disease has two clinical phases: acute and chronic. The acute phase can be asymptomatic or symptomatic with circulating trypomastigotes in peripheral blood. Decades later, 30 to 40% of patients develop a chronic phase with cardiovascular symptoms, while 10 to 15% present gastrointestinal symptoms (megaesophagus and megacolon) or remain asymptomatic (2).

Different studies have identified that the host gut microbiota composition can influence Chagas disease’s physiopathology. Robello et al. found in 2019 that the presence of T. cruzi produced alterations in the intestinal microbiota in children, producing an increased fecal abundance of Streptococcus, *Blautia*, *Butyrivibrio*, and *Roseburia* and a lower fecal abundance of *Bacteroides* (5). These changes have also been observed in murine models using bioluminescent strains of T. cruzi, highlighting a significant intestinal transit that impacts host gut microbiome. In this context, McCall et al., in 2018, observed functional changes in the intestinal chemical environment linked to conjugated linoleic acid derivatives obtained from specific members of the *Ruminococcaceae* and *Lachnospiraceae* families, thus impacting the pathophysiology of Chagas disease, particularly in chronic digestive stages. Likewise, Hossain et al. in 2020 demonstrated that high parasite burdens in the esophagus, colon, and intestine modify the composition of the microbiota (beta diversity) and metabolome (metabolites such as kynurenine and long-chain acylcarnitines). Nevertheless, an essential constraint of these studies is the use of meta-taxonomic strategies through the 16S-rRNA marker with a limited resolution at the genus level. Likewise, using murine models with different genetic and immunological backgrounds can condition the microbiome’s composition, leading to results that are not comparable between different studies (6–8).

These findings demonstrate the importance of the interaction of T. cruzi with the intestinal microbiome during infection. Also, how its proper understanding may reveal potential physiological and pathophysiological mechanisms associated with Chagas disease. Therefore, we performed a metagenomic analysis in two different mice models (BALB/c and C57BL/6) to evaluate T. cruzi infection and its impact on the microbiome. We identified taxa related to infection and functional changes associated with genes and pathways that could explain the possible metabolic alterations related to the infectious process and its interaction with the host. In addition, we were able for the first time to reconstruct MAGs from several bacterial species.

## RESULTS

### Parasitemia.

**FIG 1:**
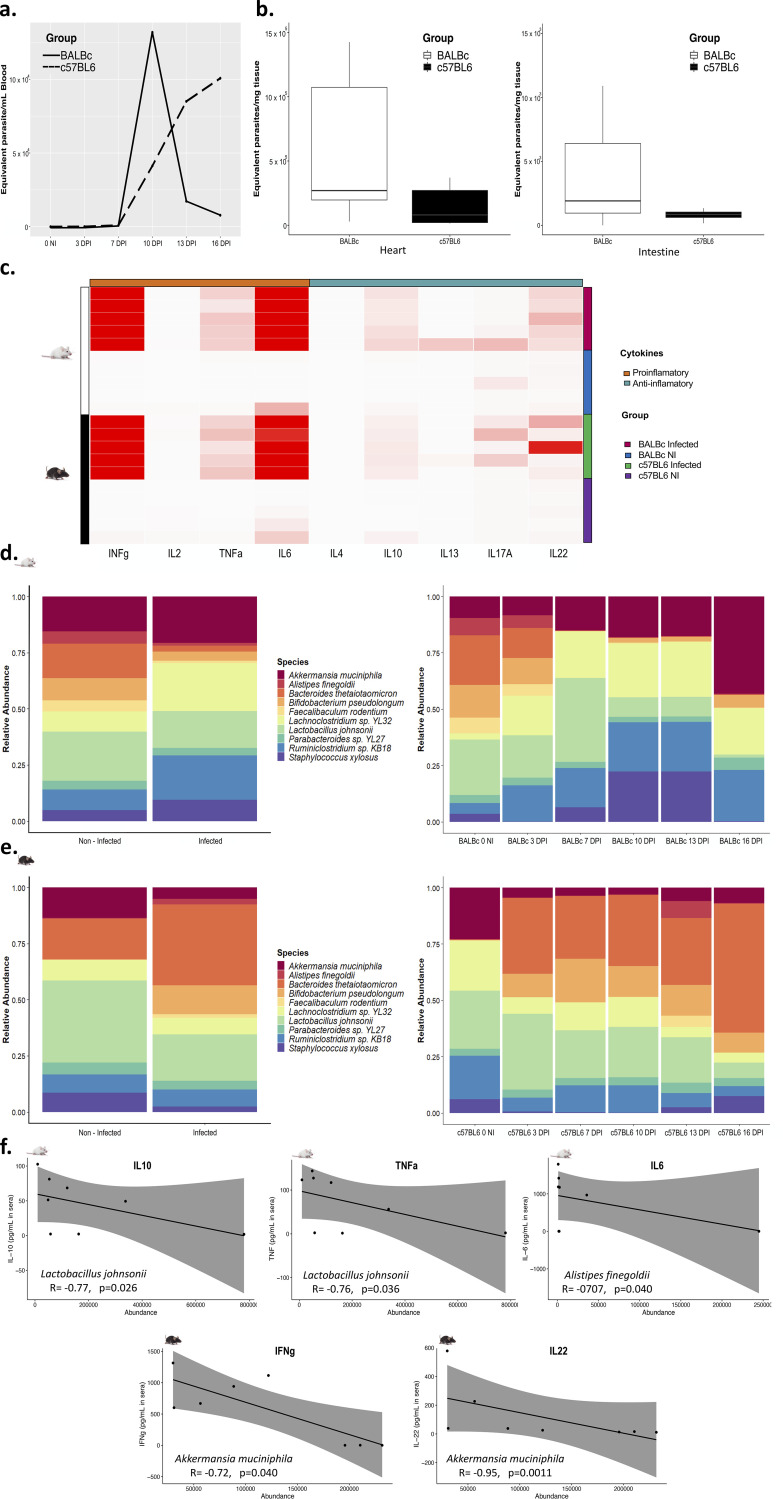
(a) Parasite burdens from total DNA were calculated by real-time PCR, and a standard curve was used to calculate the parasite equivalents/mL in blood. A solid line represents the parasite burden in BALB/c infected mice (*n* = 5), and a dashed line represents the parasite burden in C57BL/6 infected mice (*n* = 5). (b) Parasite burden in cardiac tissue and intestinal tissue. A Student *t* test was used to compare burdens in cardiac tissue, and a Wilcoxon signed-rank exact test was used to compare burdens in intestinal tissue. (c) Heatmap showing the profile of anti-inflammatory and proinflammatory cytokines according to the evaluation group. The average linkage was used as the clustering method. (d) The relative abundance of the 10 most abundant bacterial species in BALB/c mice is shown for the control (*n* = 5) and infected (*n* = 5) groups (left) and each of the measurement points (right). (e) The relative abundance of the 10 most abundant bacterial species in C57BL/6 mice is shown for the control (*n* = 5) and infected (*n* = 5) groups (left) and each of the measurement points (right). NI, noninfected; DPI, days postinfection. (f) Correlation analysis between abundance values of Lactobacillus johnsonii, Alistipes finegoldii, and Akkermansia muciniphila and levels of cytokines IL-10, TNF-α, IL-6, IFN-γ, and IL-22. A Spearman correlation test was implemented, the R coefficient, and *P* value are indicated.

At the time of euthanasia, by splenocyte cell counting, we found that both BALB/c and C57BL/6 T. cruzi-infected mice had splenomegaly. Although blood parasite burden results showed that BALB/c and C57BL/6 had a different progression in accordance with the infection, both contained detectable parasite burdens from day two and a first peak of infection at 10 DPI (Fig. 1a). At 16 days postinfection (dpi), parasite burdens in the heart and intestine showed greater amounts of the parasite in BALB/c mice than in C57BL/6 mice; nevertheless, no statistically significant differences were observed (Fig. 1b).

### Immunological profile.

We found, as expected, a proinflammatory profile for both C57BL/6 and BALB/c animals infected characterized by cytokines such as gamma interferon (IFN-γ), tumor necrosis factor alpha (TNF-α), and interleukin-6 (IL-6). Furthermore, anti-inflammatory cytokines such as IL-10 were higher in infected mice, mainly in BALB/c mice (Fig. 1c).

To identify the specific response patterns of each mouse strain, the values of the different cytokines were normalized, considering the reference values presented by noninfected mice. Therefore, the comparison between mice strains shows that BALB/c mice have higher values of the proinflammatory cytokines (IFN-γ and IL-6) and the anti-inflammatory cytokines (IL-10 and IL-4) in response to infection than C57BL/6 mice (Welch two-sample *t* test, *P* < 0.05) (see Fig. S1 in the supplemental material).

### Characterization of the gut microbiome.

After quality preprocessing and removal of host reads, approximately 14 million reads per sample were obtained for both mice strains. Of the percentage that could be taxonomically classified, more than 99% corresponded to bacteria and less than 1% to viruses. No reads corresponding to eukaryotes were identified. Metagenomic analysis showed the taxonomic identification of 4,782 bacterial species corresponding to BALB/c mice and 4,565 species to C57BL/6 mice. The predominant phyla were *Bacillota* (*Firmicutes*), *Bacteroidota* (*Bacteroidetes*), *Pseudomonadota* (*Proteobacteria*), *Actinomycetota* (*Actinobacteria*), *Verrucomicrobia*, and *Fusobacteria*. However, the mouse strains showed differences concerning the relative abundances of the different phyla (see https://github.com/gimur/Trypanosoma_cruzi-host-microbiome.git, files summary_taxonomy_BALBc_github.tsv and summary_taxonomy_BL6_github.tsv). The 10 species with the highest relative abundance in BALBc and C57BL/6 are shown in Fig. 1d and e (left panel), showing changes between the noninfected and infected groups.

Changes in the abundance of the different taxa in BALB/c mice were observed between the noninfected and the infected groups as the infection progressed (Fig. 1d, right panel). A loss in the relative abundance of specific bacterial species such as Lactobacillus johnsonii, Bacteroides thetaiotaomicron, Bifidobacterium pseudolongum, and Faecalibaculum rodentium at different time points was observed. In contrast, there was an increase in the relative abundance of other bacterial taxa, such as Akkermansia muciniphila and Staphylococcus xylosus (Fig. 1d, right panel). On the other hand, a small proportion of viruses were identified, mainly related to *Lactobacillus* phages that decrease as the infection progresses, and Mus musculus mobilized endogenous polytropic proviruses that increase with time (see Fig. S2a). These changes were progressive, more evident at 10 dpi when a first peak in the parasite burden was reached.

Since changes in the intestinal microbiota may contribute to the immune response, a correlation analysis was performed between serum cytokine concentrations and the abundance of the top 10 bacterial taxa described above (Fig. 1d and e). Spearman’s test showed significant correlations between the abundance of some bacterial taxa and the levels of different cytokines. In BALB/c mice, Lactobacillus johnsonii showed a negative correlation with the anti-inflammatory cytokine IL-10 (*P* = 0.026, *R* = −0.77) and with the proinflammatory cytokine TNF-α (*P* = 0.036, *R* = −0.76). Likewise, Alistipes finegoldii correlated negatively with the proinflammatory cytokine IL-6 (*P* = 0.040, *R* = −0.707) (Fig. 1f).

Regarding C57BL/6 mice, the differences were not as noticeable as in the case of BALB/c mice. However, some changes were evident. As the T. cruzi infectious progressed, the abundance of Bacteroides thetaiotaomicron increased, reaching its highest proportion 16 dpi. Likewise, a reduction in the relative abundance of L. johnsonii and A. muciniphila (Fig. 1e, right panel). In C57BL/6 mice, the viruses showed no changes compared to BALB/c (see Fig. S2b). Correlation analysis between serum cytokine concentrations and changes in the abundances of some intestinal microbiota taxa were statistically significant in C57BL/6 mice. A. muciniphila and IFN-γ was also evident (*P* = 0.040, *R* = −0.72), and there was also a strong negative correlation with the anti-inflammatory cytokine IL-22 (*P* = 0.0011, *R* = −0.95) (Fig. 1f).

Beta diversity estimation showed no differences in both cases (infected and noninfected mice). The analysis of nonparametric multidimensional scaling (NMDS) revealed that the overall microbiome profiles did not differ in global terms, and the changes may be related to specific taxa and not to the general microbiota of the different groups (see Fig. S2c).

### Functional analysis.

**FIG 2:**
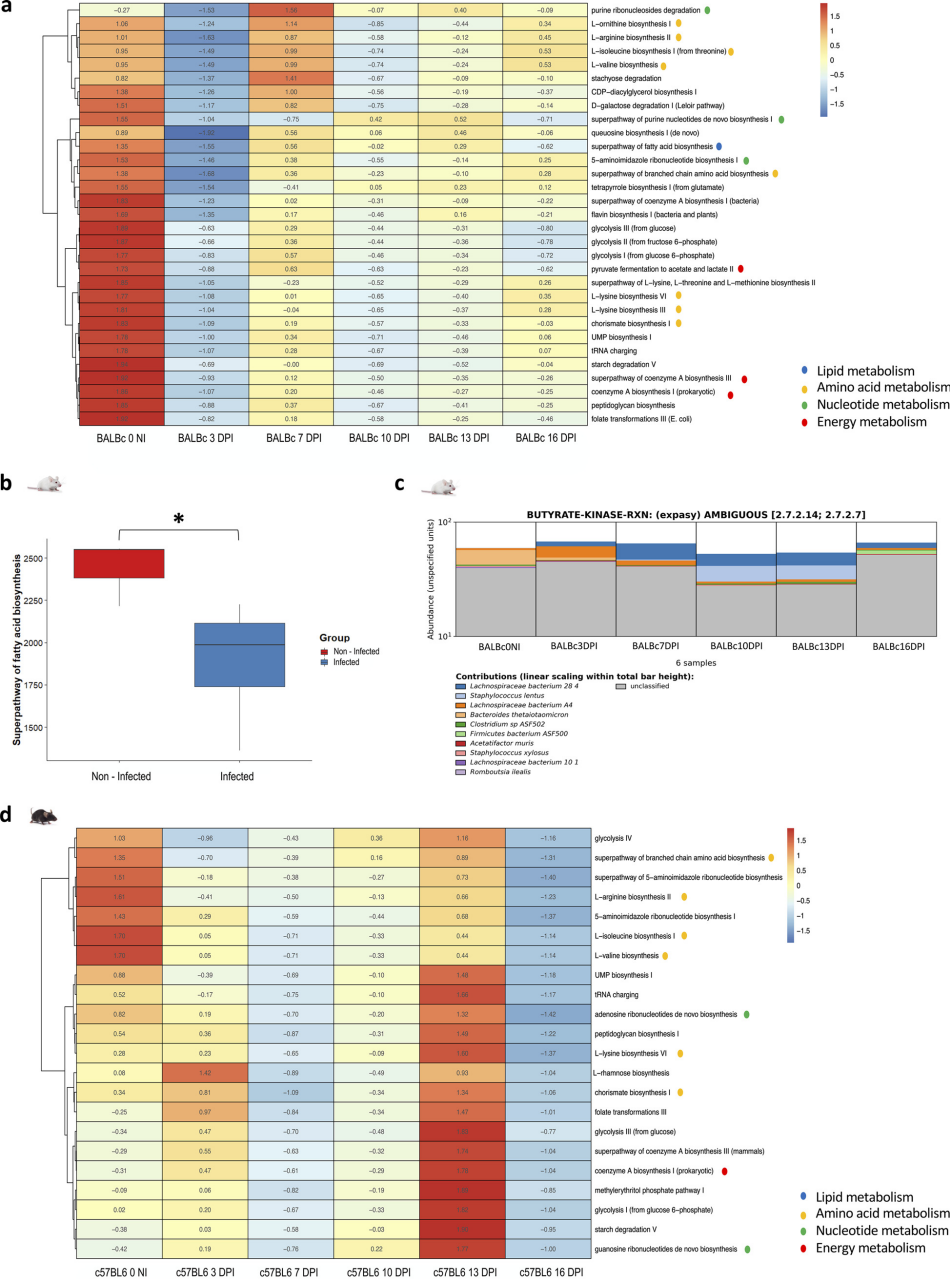
Functional analysis from metagenomic data. The main results were obtained by Humann3, and the R package, Maaslin2, using a multivariate mixed-effects model. (a and d) Metabolic pathways involving genes with differential abundances between infected and noninfected mice for BALB/c (a) and C57BL/6 (d) mice. Heatmaps were created with MicrobioSee using Ward’s linkage method for hierarchical cluster analysis. The metabolic pathways to which the different processes belong are represented by different colored dots indicated in the legend. (b) Abundance of reads related to the superpathway of fatty acid biosynthesis as observed for noninfected (NI) and infected BALB/c mice. A Student *t* test was used to compare these groups. (c) Bar plot obtained from HUMAnN 3.0 for butyrate kinase that was modified in terms of abundance of reads during T. cruzi infection. The figure includes the regrouping of bacterial species and the classification of the samples by evaluation point. *, *P* < 0.05. BALB/c infected mice, *n* = 5; BALB/c noninfected mice, *n* = 5; C57BL/6 infected mice, *n* = 5; C57BL/6 noninfected mice, *n* = 5.

The functional analysis performed by Humann3 was evaluated using the multivariate mixed-effects model implemented by Maaslin2 to identify statistically significant differences between infected and noninfected mice. It was found that changes in the abundance of specific genes involved several metabolic pathways between mice before and after infection. As in the case of the microbiota evaluation at the taxonomic level, in the functional analysis, the changes were more evident in BALB/c mice than in C57BL/6 mice (Fig. 2a and d). Genes that showed an adjusted *P* value of <0.05 were retained for analysis.

We identified 3,838 genes with statistically different abundances between noninfected and infected BALB/c mice. For C57BL/6, only 23 genes showed differences between noninfected and T. cruzi-infected mice (material available in https://github.com/gimur/Trypanosoma_cruzi-host-microbiome.git, files significant_results_Humann_BALBc.tsv and significant_results_Humann_BL6.tsv). Table 1 summarizes the major metabolic pathways, the genes involved in these pathways, the taxa associated with these genes, the false discovery rate (FDR) value, the effect size in infected mice, and the *P* and *q* statistical significance values. The genes that showed differences were related to metabolic processes such as lipid synthesis (including short-chain fatty acids [SCFAs]), branched-chain amino acid (BCAA) synthesis, purine synthesis, pyrimidine degradation, tryptophan metabolism, cobalamin metabolism, and pyruvate metabolism, among others (Fig. 2a and d). (Material is available at https://github.com/gimur/Trypanosoma_cruzi-host-microbiome.git, files PathTotal_merge_BALBc.csv and PathTotal_merge_BL6.csv).

**TABLE 1:** Functional analysis for gene and metabolic pathway prediction in infected and noninfected mice^*a*^

Metabolic pathway	Gene	Associated bacterium	FDR	Effect size in infected BALB/c mice	*P*	*q*
Lipid metabolism^*b*^	Enoyl-CoA hydratase	Bacteroides thetaiotaomicron	5.743E–02	−4.6	0.000691712	0.005358871
	Tiglyl-CoA hydratase	Bacteroides thetaiotaomicron	5.359E–03	−4.64	0.000691712	0.005358871
	Long-chain fatty acid CoA ligase	Bacteroides thetaiotaomicron	1.835E–0	−3.09	0.003273173	0.018350702
	Acetyl-CoA carboxylase	Bacteroides thetaiotaomicron	1.463E–02	−3.39	0.002282365	0.014634670
	Acyl carrier malonyltransferase	Bacteroides thetaiotaomicron	1.110E–02	−3.76	0.001535573	0.011100064
	Beta ketoacyl acyl carrier protein	Parasutterella excrementihominis	1.182E–02	−3.6	0.001679678	0.011815909
	Long-chain fatty acid CoA ligase	Acetatifactur muris	2.075E–02	2.1	0.004208897	0.020745468
Amino acid metabolism^*c*^	Isoleucine tRNA ligase	Bacteroides thetaiotaomicron	1.419E–0	−3.43	0.002190388	0.014189590
	Leucine tRNA ligase	Bacteroides thetaiotaomicron	1.566E–02	−3.3	0.002510489	0.015657878
	Tryptophan tRNA ligase	Dubosiella newyorkensis	1.557E–02	−3.31	0.002489752	0.015570720
	Tryptophan synthase	Bacteroides thetaiotaomicron	9.214E–03	−4.01	0.001215979	0.009213813
		Faecalibaculum rodentium	1.951E–02	−2.9	0.003771024	0.019505084
	Branched-chain amino acid transaminase	Acutalibacter muris	1.950E–02	−2.96	0.003768102	0.019500525
	Chorismate synthase	Parasutterella excrementihominis	2.021E–02	−2.92	0.003967455	0.020212716
Nucleotide metabolism	Ribonucleoside diphosphate reductase	Parasutterella excrementihominis	6.064E–03	−4.48	0.000787853	0.006064403
	Hypoxanthine phosphoribosyltransferase	Faecalibaculum rodentium	6.568E–03	−4.39	0.000856150	0.006567651
	Purine nucleoside phosphorylase	Bacteroides thetaiotaomicron	9.383E–03	−3.98	0.001243739	0.009382987
Biosynthetic pathway to cobalamin	Precorrin 2 dehydrogenases	Faecalibaculum rodentium	2.315E–02	−2.73	0.005085668	0.023150662
Oxidative metabolism and CoA synthesis pathway	Succinate CoA ligase ADP forming	Bacteroides thetaiotaomicron	1.430E–02	−3.42	0.002212495	0.014301774
	Dephospho-CoA kinase	Dubosiella newyorkensis	1.779E–02	−3.14	0.003048271	0.017789402
Glycolysis and fermentation pathways	Pyruvate kinase	Bacteroides thetaiotaomicron	1.743E–02	−3.16	0.002959204	0.017426635
		Dubosiella newyorkensis	2.144E–02	−2.82	0.004491714	0.021443220

a Identification of genes and associated taxa was performed using the Humann3 tool, and statistical evaluation to determine statistically significant changes was performed with Maaslin2 from a multivariate mixed-effects model. The table shows the metabolic pathway, the genes involved in this pathway, the taxa associated with these genes, the false discovery rate (FDR), the effect size in infected mice, and the *P* and *q* statistical significance values. CoA, coenzyme A.

b That is, beta-oxidation and fatty acid synthesis and metabolism.

c That is, the synthesis of SCFAs (leucine, valine, and isoleucine), the synthesis of ornithine, the synthesis of arginine, and the synthesis of chorismate (a derivative of shikimic acid metabolism and precursor of aromatic amino acids such as phenylalanine, tryptophan, and tyrosine).

Regarding lipid metabolism, potential infection-related bacteria such as Bacteroides thetaiotaomicron and Parasutterella excrementihominis were essential in lipid metabolism, specifically related to beta-oxidation and fatty acid synthesis. We found a reduction in the enzymes such as enoyl coenzyme A hydratase (enoyl-CoA hydratase), tiglyl-CoA hydratase, long-chain fatty acid CoA ligase, acetyl-CoA carboxylase carrier malonyltransferase, and beta-ketoacyl acyl carrier protein in the infected mice (Table 1). Interestingly, some bacterial taxa that increased with infection were associated with a higher abundance of genes that were reduced by the decrease in other taxa. Thus, for example, Acetatifactur muris, in infected mice, was associated with a greater abundance of genes related to long-chain fatty acid CoA ligase (Table 1).

In terms of lipid metabolism, the fatty acid synthesis super pathway showed statistically significant differences, showing a higher number of reads in noninfected mice than in infected mice (*t* test, *P* = 0.026) (Fig. 2b). This strongly correlates with specific enzymes in the functional analysis, such as butyrate kinase, which decreased with infection progress (Fig. 2c). This enzyme in noninfected BALB/c mice was mainly related to B. thetaiotaomicron. However, this taxon was reduced as infection progressed and other bacteria increased their relative abundance, mainly *Lachnospiraceae* and Staphylococcus lentus (Fig. 2c).

Regarding amino acid metabolism, both BALB/c and C57BL/6 mice showed a decrease in these metabolic pathways (Fig. 2a and d). The most relevant infection-related taxa were B. thetaiotaomicron, Dubosiella newyorkensis, *F. rodentium*, Acutalibacter muris, and *P. excrementihominis*. These bacteria, were significantly related to specific metabolic processes such as the synthesis of short-chain amino acids (leucine, valine, and isoleucine), ornithine, arginine, and synthesis of chorismate (a derivative of shikimic acid metabolism), known as an essential precursor of aromatic amino acids such as phenylalanine, tryptophan, and tyrosine (Fig. 2a and d). Specifically, BALB/c infected mice showed a lower abundance of genes related to aminoacyl-tRNA synthetases, tryptophan synthase, BCAA transaminase, and chorismate synthase (Table 1).

A decrease in purine synthesis was found in infected BALB/c and C57BL/6 mice (Fig. 2a and d). In particular, a lower abundance of genes such as ribonucleoside diphosphate reductase, hypoxanthine phosphoribosyltransferase, and purine nucleoside phosphorylase related to B. thetaiotaomicron, *P. excrementihominis*, and *F. rodentium* was observed (Table 1). In infected BALB/c mice, the abundance of genes associated with the *A. muris*-related ribonucleoside triphosphate reductase enzyme was found.

As for oxidative metabolism, the CoA synthesis pathway changes were evidenced in both BALB/c and C57BL/6 mice (Fig. 2a and d). In BALB/c infected mice, we observed a decrease in this pathway as T. cruzi infection progressed. Enzymes belonging to the tricarboxylic acid cycle were in lower abundance in infected mice, such as succinate CoA ligase ADP-forming and dephospho-CoA kinase (Table 1).

Changes in glycolysis and fermentation pathways were observed in BALB/c and C57BL/6 mice (Fig. 2a and d). In BALB/c infected mice, a decrease in the pyruvate fermentation pathway was observed as the infection progressed. In this context, a lower abundance of the genes related to the pyruvate kinase fundamental in glucose metabolism converting phosphoenolpyruvate to pyruvate was observed (Table 1).

### Metagenome-assembled genomes and pangenomic analysis.

**FIG 3:**
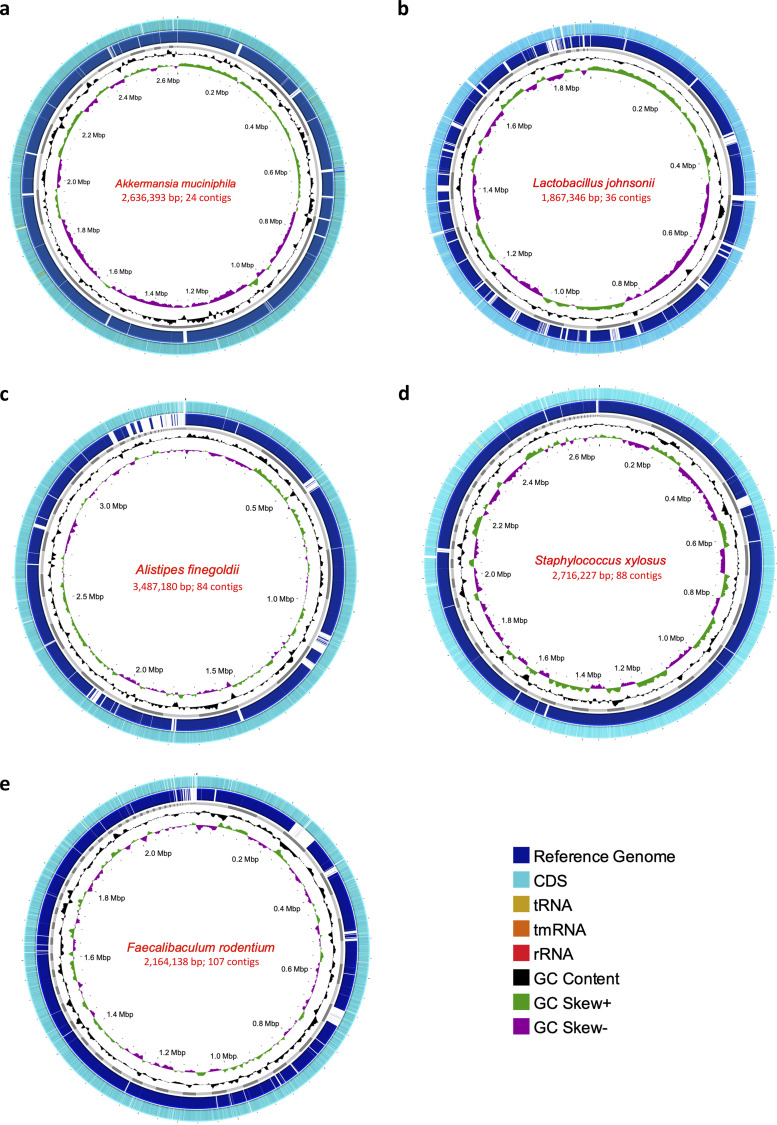
(a to e) Genomes (MAGs) reconstructed for Akkermansia muciniphila (a), Lactobacillus johnsonii (b), Alistipes finegoldii (c), Staphylococcus xylosus (d), and Faecalibaculum rodentium (e). In each of the MAGs, the size in base pairs and the resulting number of contigs of each genome obtained can be seen. For each case, the innermost ring represents the CG skew (green and purple). Subsequently, the %GC value is found (black). The next ring shows the resulting assembled genome (MAG) contigs (gray). Also, the reference genome for each genome (dark blue), which was aligned by BLAST and the white areas represent regions where no identity with the assembled MAG was found. Finally, in the outer ring, there are the coding regions obtained by Prokka, where coding regions (CDS) (light blue), tRNA (light brown), tmRNA (orange), and rRNA (red) can be identified.

From the metagenomic data for the BALB/c mice, we reconstructed eight high-quality draft metagenome-assembled genomes (MAGs). The quality statistics were evaluated with CheckM, considering completeness (>90%) and contamination (<5%) (see Table S1). The taxonomic assignment was performed with GTDB-Tk through ANI and pplacer analysis recovering genomes from L. johnsonii, *A. muciniphila*, S. lentus, *A. muris*, *F. rodentium*, *A. finegoldii*, and *S. xylosus* (Fig. 3). The MAG for B. thetaiotaomicron corresponded to a medium-quality draft since it showed contamination of 6.6% (see Table S1). Visual reconstruction of MAGs was performed in the process, including a comparative analysis against a reference genome to each case, genome annotation with Prokka, GC percentage, and GC skew (Fig. 3).

Pangenomic analysis of MAGs was made with Panaroo (see Table S2), where *A. muciniphila* showed the lowest value of the core genome with 9.6% (*n* = 743/7,645) (see Fig. S3a), *A. finegoldii* presented a core genome of 11.7%, L. johnsonii a core genome of 33.4%, *S. xylosus* a core genome of 42%, and *F. rodentium* a core genome of 59.2%, the highest such value (see Fig. S4). Aligning the extracted amino acid sequences of gene clusters of MAGs with high-quality genomes available in PATRIC indicated a close genetic congruence between each reconstructed MAGs and corresponding downloaded genomes, mainly with isolates from mice (see Fig. S3 and S4).

### Functional analysis from MAGs.

**FIG 4:**
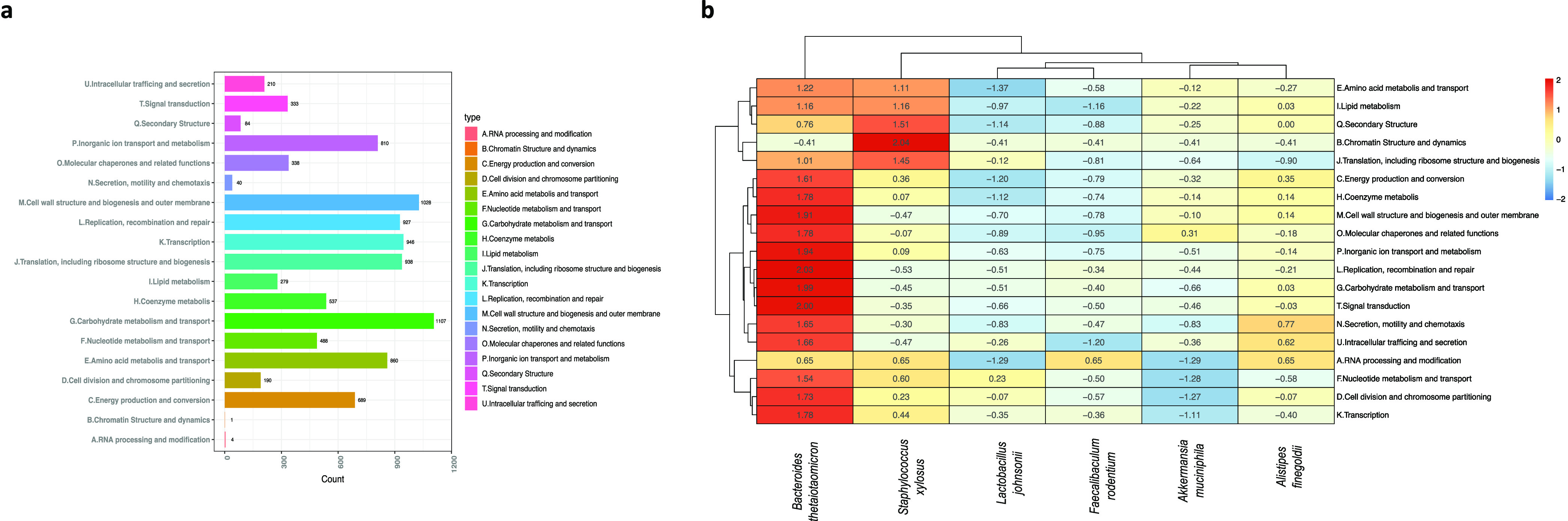
Functional analysis from MAGs. Ortholog assignment was performed using EggNOG-mapper. The results obtained here were used for clustering according to COGs of proteins in the complete set of reconstructed genomes (*n* = 6) (a) and in the different species obtained (b).

To evaluate the coding potential of the obtained MAGs, COGs were analyzed for the global data set corresponding to reconstructed MAGs. The overall results show that the highest number of genes are related to carbohydrate metabolism and transport, followed by cell wall structure, biogenesis, outer membranes, and processes associated with cell division, such as transcription, translation, and replication (Fig. 4a).

Comparative analysis concerning each of the MAGs obtained showed that B. thetaiotaomicron plays a key role in several essential metabolic processes in host physiology (Fig. 4b). Thus, genes of COGs linked to coenzyme metabolism, carbohydrate metabolism, energy metabolism, amino acid, lipid, and nucleotide metabolism were more abundant in B. thetaiotaomicron. The functional pathways (KEGG modules) in the MAGs were reconstructed from Koafm KEGG analysis through the KEGG mapper. Complete modules (functional units of genes) were found in all MAGs mainly related to essential processes such as carbohydrate metabolism, lipid metabolism, nucleotide metabolism, and amino acid metabolism.

Despite sharing the modules corresponding to common metabolic pathways among these microorganisms, specific differences were evident in some submodules between species associated with noninfected BALB/c mice such as B. thetaiotaomicron (see Fig. S5a) and species related to infected BALB/c and C57BL/6 mice such as *S. xylosus* (see Fig. S5b). It was found that in amino acid biosynthesis, B. thetaiotaomicron presents complete pathways for the synthesis of histidine mediated by the pentose pathway in its nonoxidative phase. Also, for the biosynthesis of the aromatic amino acid tryptophan from chorismate and lysine from aspartate. In terms of the biosynthesis of BCAAs, the functional analysis of the B. thetaiotaomicron genome shows the presence of the complete pathway involved in this process (see Fig. S5a). In contrast, *S. xylosus*, showed an incomplete pathway associated with histidine synthesis, where enzymes such as phosphoribosylformimino-5-aminoimidazole carboxamide were absent. Regarding the synthesis of aromatic amino acids, specifically tryptophan, *S. xylosus* presents an incomplete pathway where it lacks the enzyme anthranilate synthase, fundamental in the final step from anthranilate to indole glycerol phosphate, the precursor of tryptophan. As far as lysine synthesis is concerned, it was observed that *S. xylosus* lacks enzymes such as diaminopimelate epimerase, N-acetyldiaminopimelate deacetylase, and l,l-diaminopimelate aminotransferase, necessary for the passage from tetrahydrodipicolinate to lysine. In the specific case of BCAAs, *S. xylosus* lacks the enzyme belonging to this pathway, specifically citramalate synthase, necessary to obtain 2-methylmalate from pyruvate (see Fig. S5b).

## DISCUSSION

We identified that both mouse models showed detectable parasitemia as early as 2 dpi and burdens in both heart and intestine that were higher in BALB/c mice in both cases (Fig. 1a and b). In fact, involvement in both models was evidenced by infected mice developing splenomegaly. This is consistent with previous studies using chemiluminescent strains (6). T. cruzi generates high burdens in different portions of the gastrointestinal tract that are relevant during the acute phase, mainly at the level of the large intestine, and that can even be linked to the persistence of the parasite toward chronic digestive forms (6). As expected, the analysis of the cytokines in sera showed a proinflammatory immune response on the C57BL/6 led by IFN-γ, TNF-α, and IL-6 (Fig. 1c). In addition, the BALB/c mice revealed higher levels of anti-inflammatory cytokines such as IL-10 and IL-4 than C57BL/6 mice. This highlights the importance of this intestinal transit and how it may be related to changes in the intestinal microbiome of the infected host, a transit that deserves further investigation (6).

We observed that the structure and composition of the gut microbiome obtained through the metagenomic analyses showed the same taxa with the highest relative abundances for both animal models. These corresponded to L. johnsonii, *A. muciniphila*, B. thetaiotaomicron, B. pseudolongum, *A. finegoldii*, *F. rodentium*, and *S. xylosus*. However, these taxa showed changes in their relative abundance during the T. cruzi infection, becoming more prominent in BALB/c mice (Fig. 1d). Changes in the intestinal microbiome have previously been associated with T. cruzi infection, even in forms of digestive Chagas disease such as megacolon. Reduced relative abundances of *Bacteroides*, *Akkermansia*, and *Lactobacillus* have been reported (5, 6, 9, 10). This is consistent with our findings, demonstrating that this parasite could directly affect even other taxa that may be key in the interaction with this protozoan and that may have a direct impact on immunological and metabolic changes in the intestinal environment associated with infection.

As a result, these changes in the microbiota can potentially be associated with the immune response. Certain bacteria found in the present study, mainly strict anaerobes, are essential for the maintenance of intestinal health and proper immunoregulation (6, 9–12). This was evident with the correlation analysis between the immune response and microbiome structure, identifying a negative correlation between the reduction of L. johnsonii abundance and IL-10 in BALB/c mice (Fig. 1f). Regarding C57BL/6 mice, we found that the decrease of *A. muciniphila* correlated negatively with IL-22 and IFN-γ, explaining to some extent how *Akkermansia* may or may not favor inflammatory processes (13). Bacteria such as L. johnsonii and B. thetaiotaomicron are known due to their essential activity in the maintenance of the intestinal barrier, the inhibition of pathogen colonization, the reduction of inflammation through the regulation of the immune response, and in pivotal metabolic processes such as short-chain fatty acid (SCFA) biosynthesis (9, 13–22). T. cruzi infection directly affects the conditions of the intestinal environment causing a reduction of these populations and concomitantly, allowing the increase of taxa such as those found here. *S. xylosus* and pathobionts reported previously, such as E. faecalis, P. mirabilis, and E. coli, that are facultative anaerobes with the property of growing in this environment and associated with several inflammatory processes (23, 24). These changes favor an aerobic and inflammatory environment that metabolically may benefit the parasite. It is essential to evaluate how these taxa can represent an advantage in the infection’s establishment and progression, and whether the microbiome’s restructuring and reestablishment can potentially be a key factor in the elimination of the parasite and control of T. cruzi infection.

We corroborated the metabolic changes using functional prediction analysis from metagenomic data. The previously described disruption of the relative abundance of bacterial taxa was associated with a decrease in several metabolic processes (Fig. 2). Furthermore, BALB/c mice present a reduction in the biosynthesis of SCFAs (Fig. 2b), correlating with the taxa affected by infection and the immune response profile. An essential function of the biosynthesis of SCFAs involves the maintenance of cell differentiation and proliferation. The potential reduction of this metabolite (SCFA) may prevent proper tissue regeneration in a proinflammatory environment, thus promoting damage to the gut that allows for more efficient colonization by pathogens (25, 26). This suggests that changes in this critical metabolic pathway may favor the establishment and persistence of T. cruzi infection and be linked to the pathophysiology of the disease. However, further investigation is required to fulfill this premise.

The reconstruction of genomes from metagenomes (MAGs) supported some of the previously described findings of taxa that can potentially play a vital role in the infectious process (Fig. 3). Pangenome analysis of these MAGs revealed congruence of the reconstructed genomes with previously assembled and reported genomes. In most cases, they were found to be related to isolates obtained from mice (see Fig. S3 and S4). The analysis of the metabolic pathways performed on MAGs shows how B. thetaiotaomicron plays a fundamental role in essential metabolic processes for host physiology, such as amino acid metabolism and transport, and lipid metabolism, among others (16, 27, 28) (Fig. 4b). The observations presented above strengthen the hypothesis that the decrease of the relative abundance of this taxon may be directly implicated in the decrease of these metabolic capacities of the microbiome in T. cruzi infection. This highlights the importance of this species within the intestinal microbiome and its potential relevance in the *T. cruzi*-host-microbe interaction. On the other hand, the analysis of the MAG of a bacterium that increased with the progress of the infection such as *S. xylosus* showed that this taxon presents incomplete metabolic pathways suggesting that these bacteria are unable to supply certain biological processes (see Fig. S5). It is necessary thus to direct new studies to corroborate how bacteria such as B. thetaiotaomicron can directly modulate T. cruzi infection and how their postinfection reestablishment, for example, can favor the response to the parasite, as well as generate less tissue damage through appropriate regulation of the immune response.

It was evidenced that during the T. cruzi infection, there was a modification of the gut microbiome structure in which several taxa of interest related to the infection, such as B. thetaiotaomicron may play a key role in the interaction with the protozoan. However, with this approach, it is not possible to establish the causality and directionality of the effects evidenced at the level of the immune system and the microbiome. Regarding the above, it can be suggested that T. cruzi infection may have indirect effects on the host microbiome related to (i) the inflammatory processes of the immune response against the protozoan and (ii) with the anatomical, histological, and physiological changes associated with the pathophysiology of the disease, as in the case of megacolon, and additionally, direct effects derived directly from the presence and action of the parasite at the intestinal level.

Considering this, infection by T. cruzi and its interaction at the gut level with the host could potentially be related to several factors: (i) the generation of modifications in immune profiles that promote an increase in oxidative stress and a proinflammatory environment that favors the parasite and promote an alteration in the structure of the intestinal microbiota; (ii) the changes in this microbiota structure are directly related to functional and metabolic changes (microbiome) involving, for example, the synthesis of SCFAs, having an important relevance in the modulation of the immune response and contributing to the proinflammatory state of the infection that T. cruzi is able to evade; (iii) and, finally, T. cruzi infection may probably be generating changes in the microbiome that functionally involve biosynthesis pathways of different amino acids and nucleotide metabolism, which are potentially related to their utilization by different stages of this protozoan and are of great relevance in the life cycle of this parasite.

### Conclusions.

The changes in the intestinal microbiome identified in this study suggest that there are alterations in the immune profiles and in several essential metabolic pathways that favor the immunological and biochemical processes associated with the infection. These results provide valuable insights needed for the research on new prophylactic and therapeutic alternatives in Chagas disease. Previous studies have shown that the use of probiotics based on different species of *Lactobacillus* and *Bifidobacterium* (important in the present study) in infections by Giardia, *Cryptosporidium*, *Eimeria*, and even in systemic infections such as malaria, can favor the resolution of the infection by reducing parasitic burdens, interrupting the advance of the parasite’s life cycle, among others (29). Therefore, it is a potential area for further research and biotechnological improvement of novel therapeutic strategies for Chagas disease.

It is important to highlight, however, that a limitation of metagenomic studies is that functional analyses are based on the prediction of the genes and metabolic pathways. Therefore, it is necessary to implement transcriptomic and metabolomic studies that can directly corroborate these results and allow the identification of potential biomarkers of infection. Furthermore, the present analysis refers to the acute phase of infection; then, it is essential to also perform this evaluation in the chronic phase and thus compare the changes caused by this parasite at different times of infection and disease. In addition, it is relevant to establish the causality and directionality of the changes generated by the infection and thus determine whether the immunological effects cause changes in the microbiome or whether, on the contrary, changes in the microbiome promote alterations in the immune response profiles. Nevertheless, our study to the best of our knowledge is the first to report MAGs in hosts during an infection process with T. cruzi. Finally, this study provides a relevant overview of parasite-microbiome interactions that deserves attention and should be prioritized in the future to unveil prospective therapeutic alternatives for Chagas disease.

## MATERIALS AND METHODS

### Mice and parasites.

Female C57BL/6 and BALB/c mice from the same vendor (Jackson Laboratories), aged 5 to 8 weeks, were used. Animal experiments were performed in full compliance with the *Guide for the Care and Use of Laboratory Animals*, 8th edition (30), under a protocol approved by Baylor College of Medicine’s Institutional Animal Care and Use Committee (IACUC), assurance number and D16-00475. The Trypanosoma cruzi strain Tulahuen (ATCC 30208), a discrete typing unit (DTU) VI, was used for infection experiments. Trypomastigotes were obtained from the blood of severe combined immune-deficient (SCID) mice. Animals were injected intraperitoneally with the parasite, and trypomastigotes were isolated from blood at day 16 postinfection. All mice were maintained in the same housing conditions and on the same diet at the Baylor College of Medicine facility.

### *T. cruzi* infection.

Groups of five both BALB/c and C57BL/6 mice were kept noninfected in order to allow comparisons of the conditions evaluated throughout the follow-up. In addition, groups of five mice, BALB/c and C57BL/6, were infected with 500 blood-stage trypomastigotes of T. cruzi by intraperitoneal injection. Beginning at 3 dpi, blood was collected twice weekly throughout the study by tail vein microsampling to monitor parasitemia by quantitative PCR (qPCR) and stool for metagenome analysis. At 16 dpi, mice were euthanized, and then whole blood was collected by cardiac puncture. Lastly, different tissues (heart, and intestine) were collected and frozen at –80°C.

### Sample collection.

Fecal samples were collected (in a pool corresponding to each group) on day 0 (prior infection) and at 3, 7, 10, 13, and 16 dpi. In addition, at these time points, blood samples were taken (per mouse) for the evaluation of parasitemia by qPCR twice a week. On day 16, mice were sacrificed, and parasitemia was evaluated in cardiac and intestinal tissue.

### Parasite burdens.

To evaluate the parasite burden from blood, gut, and cardiac tissue, total DNA was isolated using a PDQeX prepGEM Universal kit (MicroGEM). Parasite burdens from total DNA were calculated by real-time PCR using the TaqMan system amplifying the satellite region of T. cruzi nuclear DNA (primers 5′-ASTCGGCTGATCGTTTTCGA-3′ and 5′-AATTCCTCCAAGCAGCGGATA-3′ and probe 5′-6-FAM-CACACACTGGACACCAA-MGB-3′), where FAM is 6-carboxyfluorescein, and MGB is minor groove binder (Life Technologies) (31, 32). To normalize the data, we used the glyceraldehyde-3-phosphate dehydrogenase (GAPDH; primers 5′-CAATGTGTCCGTCGTGGATCT-3′ and 5′-GTCCTCAGTGTAGCCCAAGATG-3′) and probe 5′-6-FAM-CGTGCCGCCTGGAGAAACCTGCC-MGB-3′ (Life Technologies) (33). The qPCRs were carried out with 4 ng of DNA from blood and 20 ng of DNA from cardiac tissue and intestine; a standard curve was used to calculate the parasite equivalents in blood, gut, and cardiac tissue (34).

### Cytokine evaluation.

The levels of IL-2, IL-4, IL-6, IL-10, IL-13, IL17A, IL-22, IFN-γ, and TNF-α were measured in sera from noninfected and infected mice; a Luminex-based assay was used, as previously described (35). The readout was performed using a MagPix Luminex instrument. Cytokine concentrations in the supernatant were calculated based on a standard curve, and for each sample, duplicate wells were averaged, and further analysis was done.

### DNA extraction for metagenome analysis.

All stool samples from animals infected and noninfected were collected and immediately stored on ice. Subsequently, DNA was extracted using MP FastDNA spin kits for soil (MP Biomedicals, Irvine, CA) (36, 37). Extracted DNA was resuspended in 50 μL of pyrogen-free water and stored at −20°C. DNA samples were shipped to Novogene for 150-bp PE sequencing by Illumina NovaSeq 6,000 to obtain 4 Gb of raw data per sample.

### Bioinformatics analysis.

**(i) Sequence processing, taxonomic assignment, and functional profiling.** From the raw reads, a quality assessment was performed by FastQC (38). Quality and adapter trimming was then conducted by Trimmomatic (39) using the parameters ILLUMINACLIP: TruSeq3-PE.fa:2:30:10:2:keepBothReads MINLEN:150 AVGQUAL:20 TRAILING:20. Mapping to the Mus musculus genome (GRCm39) was performed using the Bowtie2 tool (40) to remove reads corresponding to the host.

The Centrifuge tool was used in taxonomic assignment from the cleaned reads (41). The obtained outputs were transformed to Kraken-Report format with the Centrifuge-kreport function. Relative abundance values for each taxon identified at each time of infection were obtained from the Centrifuge report for the corresponding analysis and visualized by Pavian (42). In addition, a beta-diversity analysis was performed to compare the microbiota composition between noninfected and infected mice using NMDS.

Simultaneously, Humann3 (HMP Unified Metabolic Analysis Network) (43) was used for functional profiling, including identifying changes in gene abundance and metabolic pathways. Since this tool receives only one fastq file as input, forward and reverse reads from each sample were concatenated to generate the file. A normalization was made with RPK values to relative abundance values or “copies per million” (cpm) units through the humann_renorm_tablescript function to facilitate comparisons between samples with different sequencing depths. The default “units” of the HUMAnN microbial function are the UniRef gene families used to calculate reaction and pathway abundances. From the abundance of gene families, reconstruction of the abundance of other functional categories was performed using the humann_regroup_tablescript function. Gene family abundance values normalized by cpm to the MetaCyc reaction abundances (RXN), included with the default Humann3 facility, were regrouped. The humann_rename_table function was used to generate a readable output, and then the sample outputs were joined with the humann_join_tables function. Finally, a stratified table was obtained with humann_split_stratified_table. To evaluate the results of Humann3, the R package, Maaslin2 (44) was used to determine the association between measurement times, condition (infected – noninfected), and functional characteristics of the microbiome using a multivariate mixed-effect model. Heatmaps were created with MicrobioSee using Ward’s linkage method for hierarchical cluster analysis.

**(ii) MAGs, pangenome reconstruction, and functional analysis.** From the clean reads, the assembly process was performed using Spades, with the –meta parameter (45) and Megahit (46). MetaQuast was used to determine these assemblies’ quality statistics and to select the best assembly (47). To estimate the coverage of a bowtie2 and thus proceed to binning, reads were mapped for each sample against the corresponding assemblage. The best-quality assemblies with contigs longer than 1,500 nucleotides were binned into MAGs the using MetaBat, Maxbin, and Concoct tools, followed by refinement with DASTool (48). The quality of these final bins was evaluated with CheckM (48–51). Taxonomic assignment of these good-quality bins was performed with Genome Taxonomy Database GTDB-Tk (52). Bins with completeness >90% and contamination <5% that were taxonomically assigned were treated as MAGs for further analysis. MAGs for Akkermansia muciniphila, Lactobacillus johnsonii, Alistipes finegoldii, Staphylococcus xylosus, Faecalibaculum rodentium, and Bacteroides thetaiotaomicron were obtained. The MAGs were annotated using Prokka (53). The process was implemented for visual genome reconstruction. In this case, a BLAST alignment was performed with the default parameters with the reference genome corresponding to each previously defined species, and further downloaded from the NCBI: NZ_AP021898.1, Akkermansia muciniphila; NZ_CP062068.1, Lactobacillus johnsonii; NZ_JADMWR010000010.1, Alistipes finegoldii; NZ_CP008724.1, Staphylococcus xylosus; NZ_CP011391.1, Faecalibaculum rodentium; and NZ_CP040530.1, Bacteroides thetaiotaomicron. The .gff annotation files obtained for each case were also included in this reconstruction, where the GC percentage and the GC Skew with the default parameters were determined.

The .gff files obtained from the annotation also were used to conduct a pangenome analysis using Panaroo tool (54), considering an identity of 99% and their presence in the compared genomes of 99% (–clean-mode strict -a core –aligner mafft –core_threshold 0.99). For this purpose, the complete and good-quality genomes available in PATRIC (Pathosystems Resource Integration Center) were downloaded: 96 genomes of Akkermansia muciniphila, 15 of Lactobacillus johnsonii, 18 of Alistipes finegoldii, 20 of Staphylococcus xylosus, and 4 of Faecalibaculum rodentium. The results were used to generate phylogenetic trees in ITol (55), based on alignments obtained from the core genome using Panaroo and the .newick files generated from these alignments. For the graphical representation of the core genome was used Phandango (56).

The Kyoto Encyclopedia of Genes and Genomes (KEGG) was used to identify the metabolic capacities present in MAGs (57, 58). KofamKOALA was used to assign K numbers to the sequence data by HMMER/HMMSEARCH against Kofam (a customized HMM database of KEGG Orthologs [KOs]). The outputs were used for visual reconstructions of metabolic pathways using the KEGG Mapper. Complementarily, an evaluation was performed for annotation through ortholog assignment using EggNOG-mapper (59). Finally, the results were used for clustering according to Clusters of Orthologous Groups (COGs) of proteins (60).

### Statistical analysis.

Statistical analyses were carried out using R software (61). For continuous values (parasite burdens, relative abundance, cytokine levels, and abundance of gene reads), normality hypotheses were evaluated using the Kolmogorov-Smirnov and Shapiro-Wilk tests. A comparison of the values of numerical variables (parasite burdens, relative abundances, cytokine levels, and abundance of gene reads) was performed between study groups (noninfected and infected mice) and between time points of infection. For this purpose, parametric tests (Student *t* test for comparisons between two groups) and nonparametric tests (Wilcoxon signed-exact-ranks test for comparisons between two groups and Kruskal-Wallis test for comparisons between multiple groups with the corresponding Dunn’s test for *post hoc* multiple comparisons adjusted with the Bonferroni method) were used. Likewise, to evaluate the correlation between the numerical variables corresponding to abundances of the different taxa and the pg/mL serum levels of the cytokines, Spearman’s nonparametric test was used. All significance tests, whether parametric or nonparametric, were two tailed, and *P* values of <0.05 were considered statistically significant.

### Ethics approval and consent to participate.

Animal experiments were performed in full compliance with the *Guide for the Care and Use of Laboratory Animals*, 8th edition (30), under a protocol approved by Baylor College of Medicine’s IACUC, under assurance number D16-00475.

### Data availability.

Sequence data supporting the conclusions of this article are available in the SRA database under the BioProject PRJNA915351. Code for generating the figures and the analysis is available at https://github.com/gimur/Trypanosoma_cruzi-host-microbiome.git.
