# Supplementary material for: Microbiome Alterations Driven by Trypanosoma cruzi Infection in Two Disjunctive Murine Models
Source: Microbiol Spectr. 2023 May 4;11(3):e00199-23. doi: 10.1128/spectrum.00199-23 (PMC10269900; doi:10.1128/spectrum.00199-23)
Supplement: Supplemental file 1 — Supplemental material. Download spectrum.00199-23-s0001.pdf, PDF file, 1.3 MB [file spectrum.00199-23-s0001.pdf]

Supplemental Material

Figure S1. Evaluation of the immune profile in response to *Trypanosoma cruzi* infection. (a) Anti-inflammatory and (b) proinflammatory cytokines evaluated. Welch Two Sample t-test was performed. * $p < 0.05$. BALBc infected mice, n =5; BALBc non-infected mice, n =5; c57BL6 infected mice, n =5; c57BL6 non-infected mice, n =5.

Figure S2. Relative abundance of the 10 most abundant viruses identified in (a) BALBc and (b) c57BL6 mice for each of the measurement points. NI = non-infected; DPI = days post-infection. (c) Beta diversity analysis was performed by non-parametric multidimensional scaling (NMDS) to compare the microbiota composition between controls and infected BALBc (left) and c57BL6 (right) mice. BALBc infected mice, n =5; BALBc non-infected mice, n =5; c57BL6 infected mice, n =5; c57BL6 non-infected mice, n =5.

Figure S3. Pangenome analysis for (a) *Akkermansia muciniphila* and (b) *Lactobacillus johnsonii*. Complete and good-quality genomes available in PATRIC were downloaded: 96 genomes of *Akkermansia muciniphila* and 15 of *Lactobacillus johnsonii.* Phylogenetic trees were made in ITol (left) where the source of each isolate was defined according to the available information. The bootstrap value is described between 0 and 1. The branch corresponding to the reconstructed MAG for each case is highlighted in red. The heatmap (right) represents the core genome for the different isolates (including the reconstructed MAGs).

Figure S4. Pangenome analysis for (a) *Alistipes finegoldii*, (b) *Staphylococcus xylosus,* and (c) *Faecalibaculum rodentium*. Complete and good-quality genomes available in PATRIC were downloaded: 18 of *Alistipes finegoldii*, 20 of *Staphylococcus xylosus,* and 4 of *Faecalibaculum rodentium*. Phylogenetic trees were made in ITol (left) where the source of each isolate was defined according to the available information. The bootstrap value is described between 0 and 1. The branch corresponding to the reconstructed MAG for each case is highlighted in red. The heatmap (right) represents the core genome for the different isolates (including the reconstructed MAGs).

Figure S5. Reconstruction of the amino acid synthesis pathway for the genomes of (a) *Bacteroides thetaiotaomicron* and (b) *Staphylococcus xylosus,* obtained by KEGG mapper from the functional analysis by Koafm. This map presents a modular architecture of the biosynthesis pathways of twenty amino acids, which may be viewed as consisting of the core part and its extensions. The core part is the KEGG module for conversion of three-carbon compounds from glyceraldehyde-3P to pyruvate, together with the pathways around serine and glycine. This KEGG module is the most conserved one in the KEGG MODULE database and is found in almost all the completely sequenced genomes. The extensions are the pathways containing the reaction modules RM001, RM033, RM032, and RM002 for the biosynthesis of branched-chain amino acids (left) and basic amino acids (bottom), and the pathways for biosynthesis of histidine and aromatic amino acids (top right). It is interesting to note that the so-called essential amino acids that cannot be synthesized in humans and other organisms generally appear in these extensions. Furthermore, the bottom extension of basic amino acids appears to be most divergent containing multiple pathways for lysine biosynthesis and multiple gene sets for arginine biosynthesis. Enzymes and metabolites

encoded in each genome are shown in green and the red boxes represent the absence of certain enzymes lacking in the *Staphylococcus xylosus* genome.

Table S1. CheckM statistics from MAGs were obtained with their corresponding taxonomic assignment made by GTDB-Tk.

Table S2. Summary statistics resulting from pangenome analysis. The results obtained by Roary and Panaroo are shown.

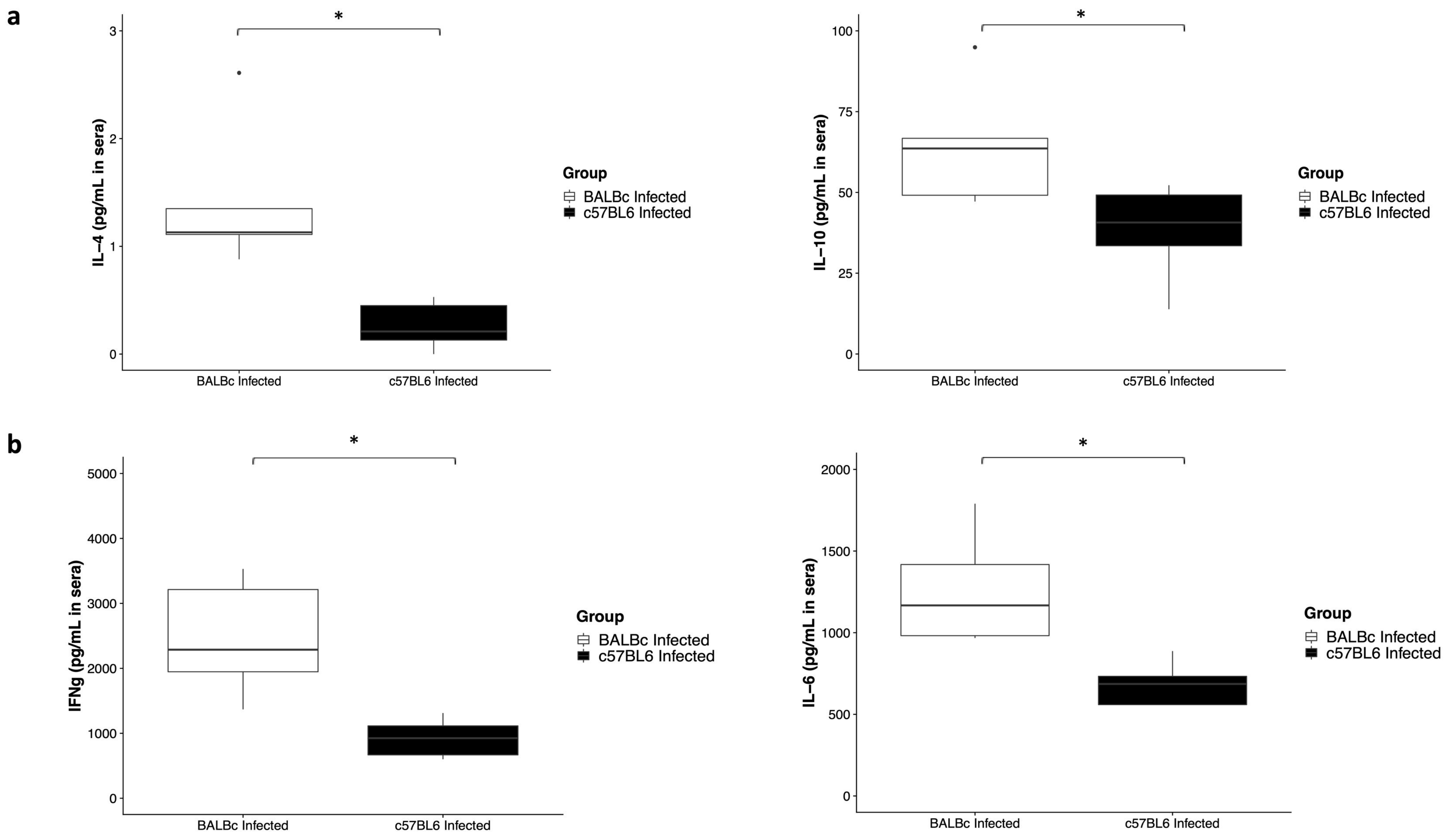

a
b
*
IL−4 (pg/mL in sera)
IL−10 (pg/mL in sera)
IFNg (pg/mL in sera)
IL−6 (pg/mL in sera)
BALBc Infected
c57BL6 Infected
Group
BALBc Infected
c57BL6 Infected

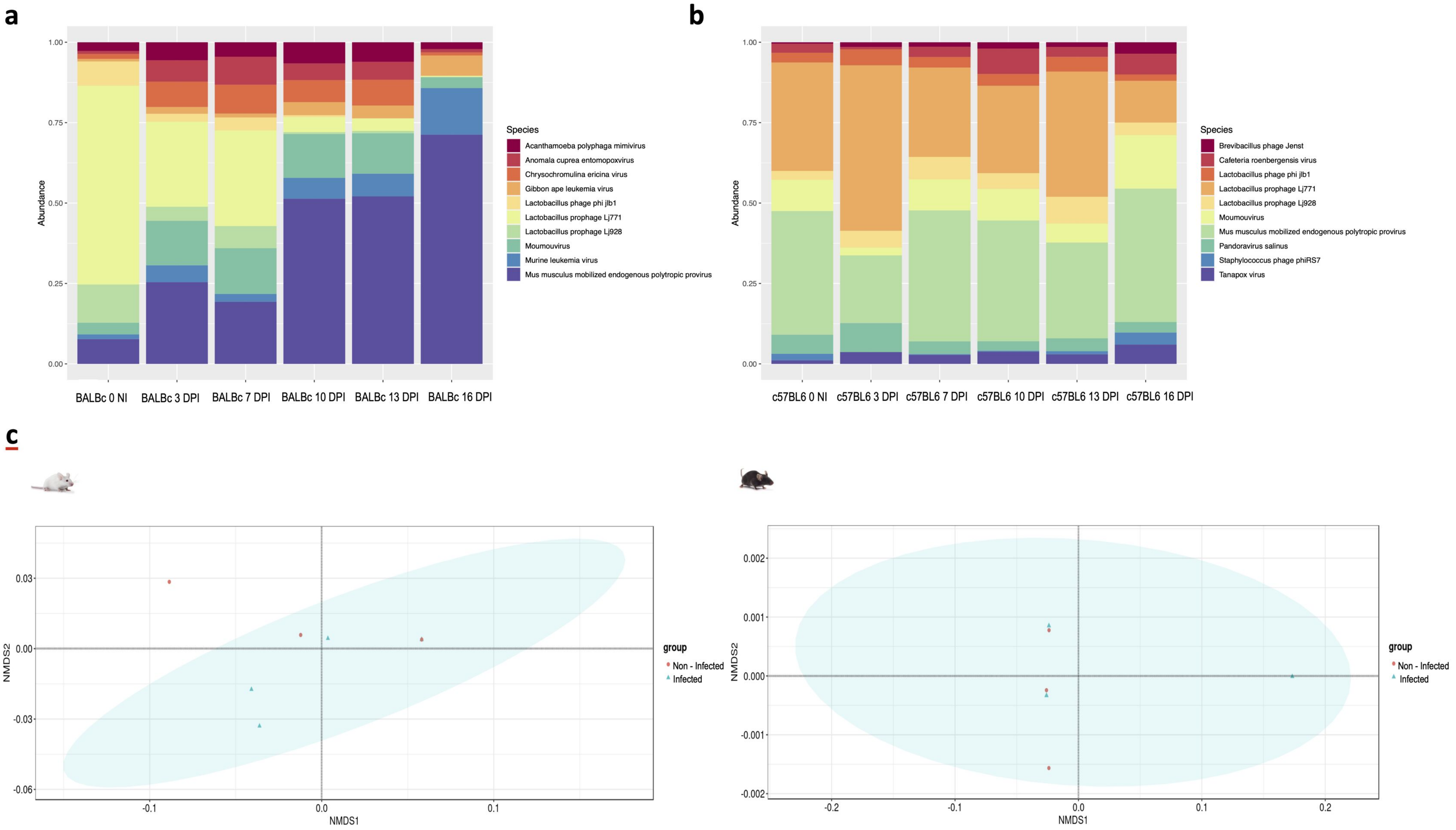
a
Abundance
1.00
0.75
0.50
0.25
0.00
BALBc 0 NI
BALBc 3 DPI
BALBc 7 DPI
BALBc 10 DPI
BALBc 13 DPI
BALBc 16 DPI
Species
Acanthamoeba polyphaga mimivirus
Anomala cuprea entomopoxvirus
Chrysochromulina ericina virus
Gibbon ape leukemia virus
Lactobacillus phage phi jlb1
Lactobacillus prophage Lj771
Lactobacillus prophage Lj928
Moumouvirus
Murine leukemia virus
Mus musculus mobilized endogenous polytropic provirus
b
Abundance
1.00
0.75
0.50
0.25
0.00
c57BL6 0 NI
c57BL6 3 DPI
c57BL6 7 DPI
c57BL6 10 DPI
c57BL6 13 DPI
c57BL6 16 DPI
Species
Brevibacillus phage Jenst
Cafeteria roenbergensis virus
Lactobacillus phage phi jlb1
Lactobacillus prophage Lj771
Lactobacillus prophage Lj928
Moumouvirus
Mus musculus mobilized endogenous polytropic provirus
Pandoravirus salinus
Staphylococcus phage phiRS7
Tanapox virus
c
NMDS2
0.03
0.00
-0.03
-0.06
-0.1
0.0
0.1
NMDS1
group
Non - Infected
Infected
NMDS2
0.002
0.001
0.000
-0.001
-0.002
-0.2
-0.1
0.0
0.1
0.2
NMDS1
group
Non - Infected
Infected

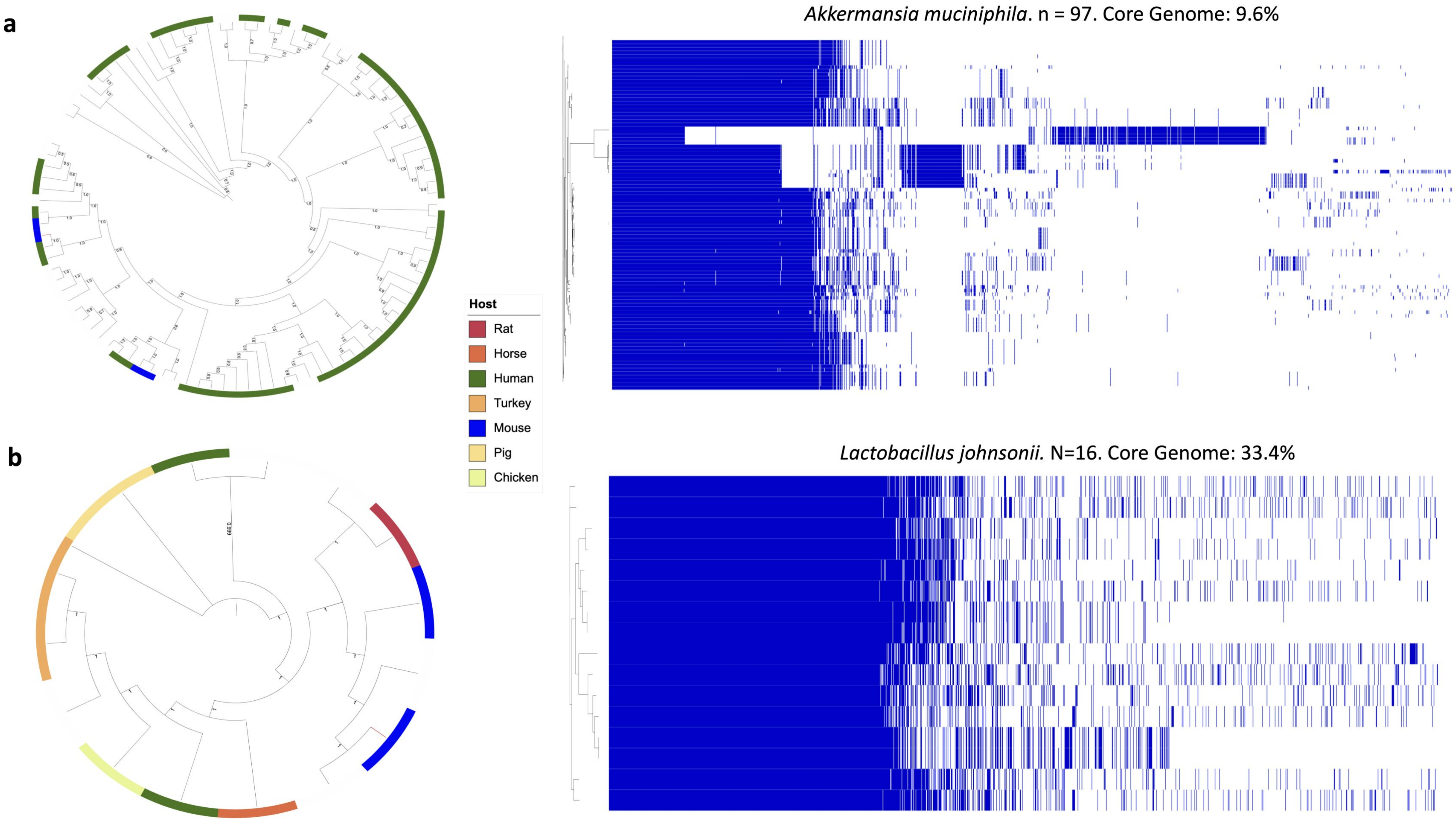
a
Akkermansia muciniphila. n = 97. Core Genome: 9.6%
b
Lactobacillus johnsonii. N=16. Core Genome: 33.4%
Host
Rat
Horse
Human
Turkey
Mouse
Pig
Chicken

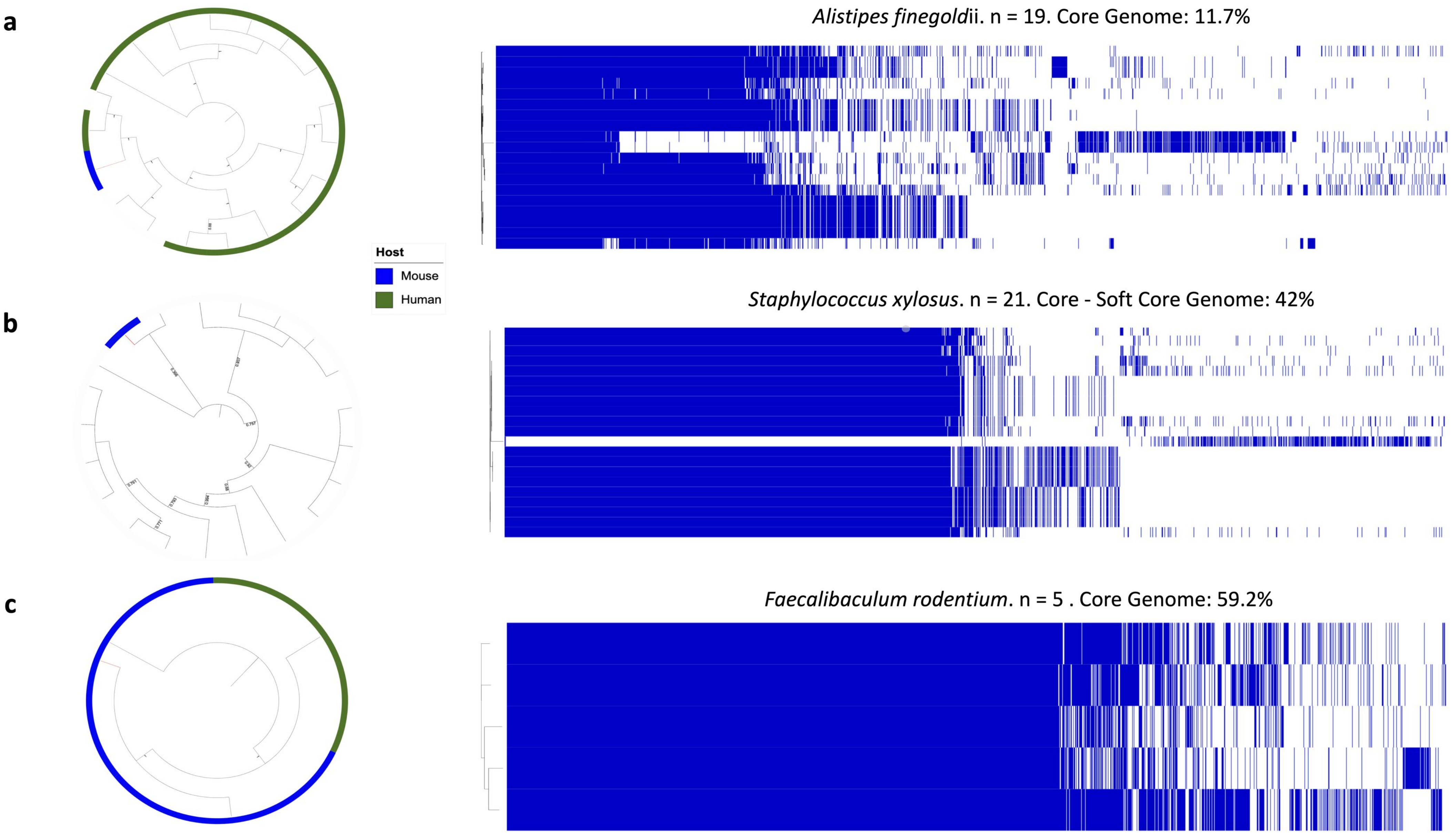
a
Alistipes finegoldii. n = 19. Core Genome: 11.7%
Host
Mouse
Human
b
Staphylococcus xylosus. n = 21. Core - Soft Core Genome: 42%
c
Faecalibaculum rodentium. n = 5 . Core Genome: 59.2%

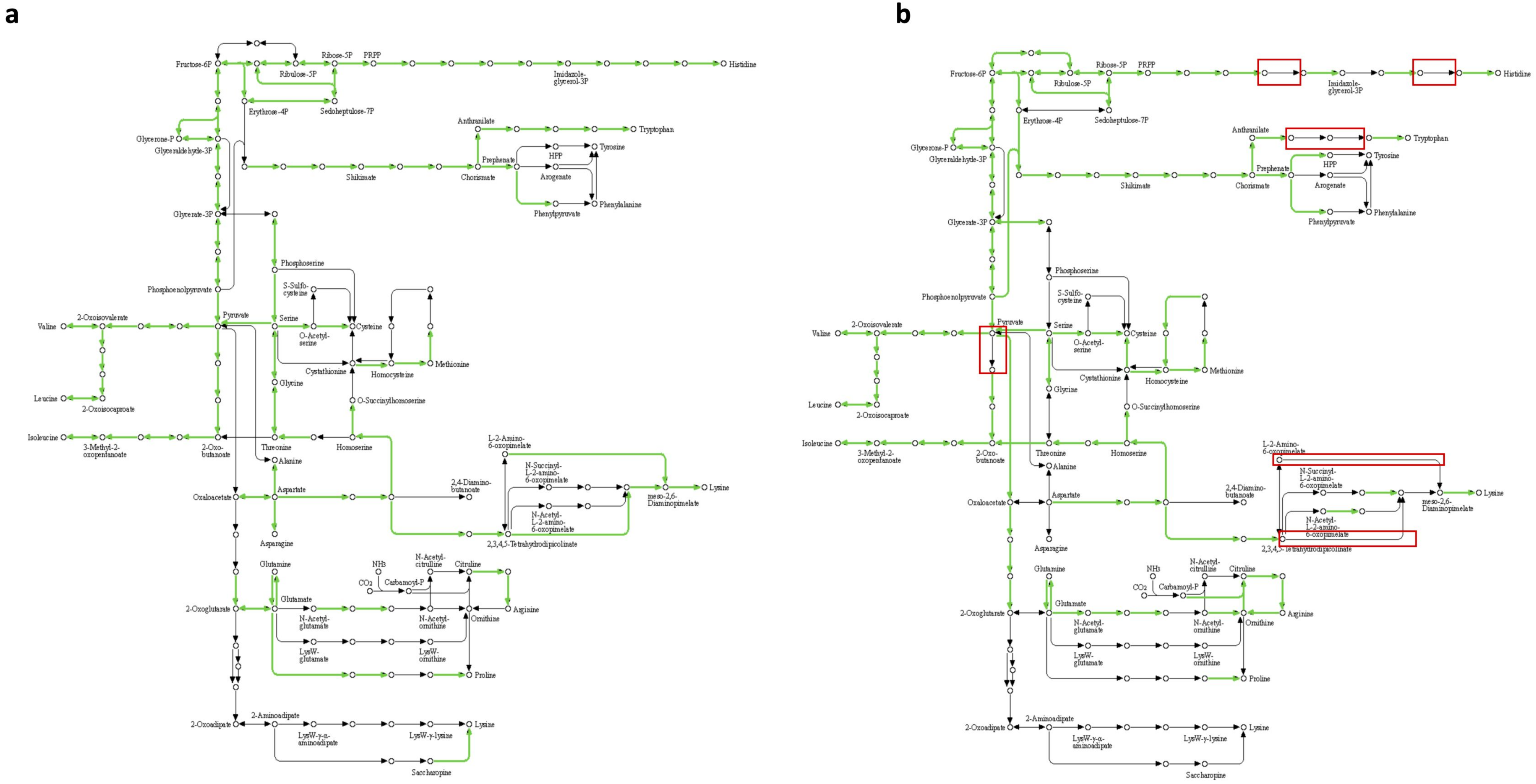
a
b
Fructose-6P
Ribose-5P
PRPP
Ribulose-5P
Imidazole-glycerol-3P
Histidine
Erythrose-4P
Sedoheptulose-7P
Glycerone-P
Glyceraldehyde-3P
Anthranilate
Tryptophan
Prephenate
HPP
Tyrosine
Shikimate
Chorismate
Arogenate
Phenylalanine
Phenylpyruvate
Glycerate-3P
Phosphoserine
S-Sulfo-cysteine
Phosphoenolpyruvate
Pyruvate
Serine
Cysteine
2-Oxoisovalerate
Valine
O-Acetyl-serine
Cystathionine
Homocysteine
Methionine
Glycine
O-Succinylhomoserine
Leucine
2-Oxoisocaproate
Isoleucine
3-Methyl-2-oxopentanoate
2-Oxo-butanoate
Threonine
Homoserine
Alanine
L-2-Amino-6-oxopimelate
N-Succinyl-L-2-amino-6-oxopimelate
2,4-Diamino-butanoate
Oxaloacetate
Aspartate
meso-2,6-Diaminopimelate
Lysine
N-Acetyl-L-2-amino-6-oxopimelate
Asparagine
2,3,4,5-Tetrahydrodipicolinate
Glutamine
NH3
N-Acetyl-citrulline
Citrulline
CO2
Carbamoyl-P
Glutamate
2-Oxoglutarate
N-Acetyl-glutamate
N-Acetyl-ornithine
Ornithine
Arginine
LysW-glutamate
LysW-ornithine
Proline
2-Oxoadipate
2-Aminoadipate
LysW-γ-α-aminoadipate
LysW-γ-lysine
Saccharopine
